# Potential Therapeutic Strategies to Overcome Acquired Resistance to BRAF or MEK Inhibitors in BRAF Mutant Cancers

**DOI:** 10.18632/oncotarget.262

**Published:** 2011-04-19

**Authors:** Ryan B. Corcoran, Jeffrey Settleman, Jeffrey A. Engelman

**Affiliations:** ^1^ Massachusetts General Hospital Cancer Center, Boston, MA 02129, USA; ^2^ Department of Medicine, Harvard Medical School, Boston, MA 02115, USA; ^3^ Genentech, South San Francisco, CA 94080, USA

**Keywords:** BRAF, MEK, acquired resistance, melanoma, colorectal cancer, NRAS, IGF1R, PDGFR

## Abstract

Recent clinical trials with selective inhibitors of the BRAF and MEK kinases have shown promising results in patients with tumors harboring BRAF V600 mutations. However, as has been observed previously with similarly successful targeted therapies, acquired resistance to these agents is an emerging problem that limits their clinical benefit. Several recent studies from our laboratory and others have investigated the causes of acquired resistance to BRAF and MEK inhibitors, and multiple resistance mechanisms have been identified. Here, we review these mechanisms and suggest that they can be broadly grouped into two main classes: ERK-dependent and ERK-independent. We also propose distinct therapeutic strategies that might be employed to overcome each class of acquired resistance.

## THE RAF-MEK-ERK PATHWAY AND CANCER

The RAF-MEK-ERK pathway regulates many important cellular processes (reviewed in [1-3]). Classically, signaling through this pathway is driven by growth factor receptor activation of RAS family GTPases, including HRAS, KRAS, and NRAS. Activated RAS proteins can complex with and activate members of the RAF kinase family—ARAF, BRAF, and CRAF. Once activated, RAF kinases phosphorylate and activate the MEK (mitogen-activated or extracellular signal-related protein kinase kinase) kinases, MEK1 and MEK2, which subsequently phosphorylate and activate ERK1 and ERK2. ERK (extracellular signal-regulated kinase) kinases phosphorylate a number of substrates with critical roles in regulating gene expression, proliferation, and cell survival.

Consistent with its role as a key regulatory pathway for cell survival and proliferation, RAF-MEK-ERK signaling is frequently dysregulated in cancer. RAF-MEK-ERK signaling can be driven by aberrant activation of growth factor receptor tyrosine kinases (RTKs) or by oncogenic mutations of intracellular components of this pathway. Indeed, activating RAS mutations (occurring most often in KRAS, followed by NRAS) are the most common oncogenic mutations observed thus far in human cancer [4]. Similarly, activating BRAF mutations are found in ~7% of human cancers, with particularly high frequency in melanoma (50-70%), papillary thyroid cancers (40%), and colorectal cancers (10-15%) [5]. Over 95% of BRAF mutations are point mutations involving valine 600 (V600) with more than 90% of these mutations encoding a substitution of V600 with a glutamic acid (V600E). BRAF V600 mutations lead to constitutive BRAF kinase activity and can promote oncogenesis in mouse tumor models [6-9]. As a result, considerable effort has been devoted to the development of therapeutic strategies directed against mutant BRAF and its key effectors.

## BRAF AND MEK INHIBITORS IN THE TREATMENT OF BRAF MUTANT CANCERS

Preclinical data has demonstrated that most BRAF mutant human tumor-derived cell lines are exquisitely sensitive to pharmacologic inhibition of RAF-MEK-ERK signaling. Thus, selective BRAF and MEK kinase inhibitors potently block cell proliferation and induce apoptosis in BRAF mutant cancer models and show high selectivity for cancers with BRAF mutations [10-12]. As a result, several BRAF and MEK inhibitors are currently in clinical development. Consistent with preclinical observations, while early clinical trials with RAF and MEK inhibitors in unselected patient populations produced few responses [13-15], recent clinical trials have focused on administering these agents specifically to patients with BRAF mutant tumors and have produced encouraging results. In a Phase I/II trial of the selective BRAF inhibitor PLX4032 in melanoma patients harboring the BRAF V600 mutation, 81% of patients achieved an objective response (defined as a reduction in tumor size of at least 30%) [16]. Interestingly, in a small study of 25 BRAF V600 mutant colorectal cancer patients treated with PLX4032, only 1 patient (5%) achieved a partial response, with an additional 4 patients (20%) achieving stable disease, suggesting that different tumor types may exhibit varied dependence on mutant BRAF [17]. Another selective BRAF inhibitor GSK2118436 produced a 60% response rate in patients with BRAF V600 mutant melanomas [18]. In early studies, the MEK inhibitor GSK1120212 produced a 21% response rate in BRAF V600 mutant melanoma patients [19]. While this response rate was lower than that observed for the two selective BRAF inhibitors mentioned above, an additional 54% of patients achieved stable disease with GSK1120212, suggesting that MEK inhibitors may still play an important clinical role in the treatment of BRAF mutant cancers.

One potential reason that BRAF inhibitors have shown higher response rates than MEK inhibitors in BRAF V600 mutant melanomas relates to a unique characteristic of RAF signaling that was elucidated during the past year by several elegant studies [20-22]. These groups found that while BRAF inhibitors potently inhibited ERK phosphorylation in BRAF V600 mutant cells, BRAF inhibitors failed to inhibit, and in some cases paradoxically increased, levels of phosphorylated ERK (P-ERK) in cells with wild-type BRAF. Activation of P-ERK by BRAF inhibitors in BRAF wild-type cells was more pronounced in cells with active RAS, either due to RAS mutation or to activation of RAS by upstream signaling components, such as RTKs. While mutant BRAF signals as a monomer, these groups found that in the presence of active RAS, wild-type BRAF forms homodimers or heterodimers with other RAF proteins, such as CRAF. When a BRAF inhibitor binds to one member of a RAF dimer, it blocks the catalytic activity of the protein to which it is bound, but it also induces transactivation of the inhibitor-free member of the RAF dimer, leading to an increase in catalytic activity and enhanced phosphorylation of the RAF substrate MEK. As a result, P-ERK inhibition by BRAF inhibitors is restricted to BRAF mutant cells, enabling a high dose of BRAF inhibitor to be administered without causing the toxic effects of ERK inhibition in normal tissues. Conversely, MEK inhibitors inhibit ERK phosphorylation in all cells, potentially leading to toxicity caused by suppression of P-ERK in normal tissues, and consequently limiting the dose that can be administered in patients. In other words, the narrower therapeutic window of MEK inhibitors may explain why BRAF inhibitors have produced higher response rates than MEK inhibitors in patients with BRAF mutant tumors.

While the initial response rates seen in BRAF mutant melanomas with BRAF and MEK inhibitors are encouraging, previous experience with similarly effective targeted therapies predicts that acquired drug resistance will be a major factor limiting the clinical benefit of these agents. Indeed, despite dramatic initial responses, the median time to progression of patients treated with PLX4032 was 7 months [16]. Understanding the mechanisms by which patients' tumors acquire resistance to targeted therapies can potentially lead to strategies to overcome resistance. Accordingly, significant effort has been devoted recently to studying acquired resistance to BRAF and MEK inhibitors in BRAF mutant cancers.

## ACQUIRED RESISTANCE TO BRAF AND MEK INHIBITORS

Preclinical modeling of acquired drug resistance has been a useful tool for predicting the resistance mechanisms that emerge in patients receiving targeted cancer therapies. Previously, this approach has predicted the resistance mechanisms that occur clinically in many instances, including erlotinib-resistance in EGFR mutant lung cancer, imatinib-resistance in BCR-ABL translocated leukemia, resistance to Smoothened inhibitors in Patched1-deficient medulloblastoma, and resistance to ALK inhibitors in ALK-translocated lung cancers [23-28]. In several cases, these findings have led to strategies to overcome resistance, which are now being used in the clinic.

**Figure 1:**
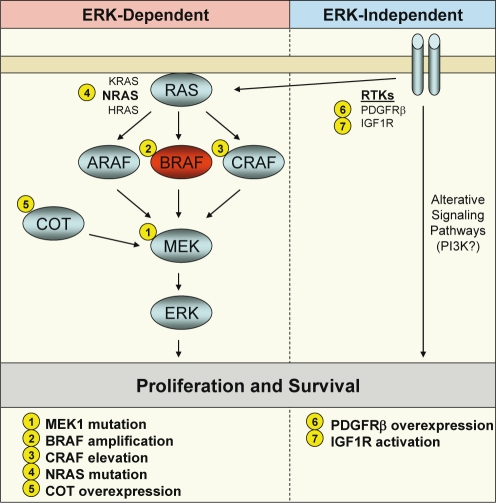
Mechanisms of acquired resistance to BRAF and MEK inhibitors in BRAF mutant cancers A schematic of the RAF-MEK-ERK signaling pathway is shown with BRAF in red. Alterations of signaling pathway components leading to resistance to BRAF or MEK inhibitors are indicated by number. Resistance mechanisms classified as ERK-dependent are shown in the left panel, and mechanisms classified as ERK-independent are shown in the right panel.

To date, preclinical modeling has identified multiple potential mechanisms of acquired resistance to BRAF or MEK inhibitors (Fig. 1), and some of these mechanisms have been validated clinically. In general, these resistance mechanisms, reviewed below, fall into two broad categories—those that retain their dependence on ERK signaling and those that do not. We will refer to these two classes as ERK-dependent and ERK-independent mechanisms of acquired resistance.

## ERK-DEPENDENT MECHANISMS OF ACQUIRED RESISTANCE

The vast majority of acquired resistance mechanisms to BRAF or MEK inhibitors that have been identified to date lead to reactivation of ERK signaling despite the presence of inhibitor. This finding underscores the importance of ERK signaling for the continued proliferation and survival of BRAF mutant cancer cells. In general, mechanisms of acquired resistance to targeted therapies commonly employ either mutation or amplification of the drug target itself or alterations which do not involve the drug target but that activate parallel or downstream signaling pathways to circumvent the activity of the drug [23-34]. In resistant BRAF mutant tumor models, examples of each of these common mechanisms has been identified in resistant BRAF mutant tumor models that lead to reactivation of ERK signaling.

### Point mutations in MEK1

Emery et al identified a MEK1 point mutation in a resistant focus of disease in a patient with V600E mutant melanoma who had originally responded to the MEK inhibitor AZD6244 [35]. Through a mutagenesis screen of MEK1, the authors also identified several additional point mutations that could potentially lead to MEK inhibitor resistance. The majority of these mutations clustered within or near the allosteric drug-binding pocket and were hypothesized to interfere with inhibitor binding. Other point mutations were identified outside of the drug-binding pocket and were thought to influence intrinsic MEK kinase activity or to affect protein conformation. These point mutations severely attenuated the ability of MEK inhibitors to inhibit ERK phosphorylation. Some MEK1 mutations, including the P124L mutation identified in a patient's AZD6244-resistant melanoma, also led to cross-resistance to BRAF inhibitors, presumably by causing activation of MEK downstream of BRAF. Interestingly, later work by this same group identified a different MEK1 point mutation (C121S) in a post-relapse biopsy from a patient with clinically acquired resistance to the BRAF inhibitor PLX4032, demonstrating that MEK1 mutations can arise as a potential mechanism of acquired resistance to BRAF inhibitors as well [36]. Interestingly, although the MEK1 P124L point mutation conferred resistance to MEK or BRAF inhibitors alone, the combination of a MEK inhibitor and BRAF inhibitor could overcome resistance in this setting.

To date, no secondary BRAF mutations have been identified in BRAF inhibitor-resistant pre-clinical models or in biopsies from patients with clinically acquired BRAF inhibitor resistance. Nazarian et al screened twelve tumor biopsies from patients with clinically acquired resistance to PLX4032 and did not observe any secondary BRAF mutations [37]. “Ultra-deep” sequencing of these tumors also failed to reveal any evidence of secondary BRAF mutations. Notably, the BRAF T529 “gatekeeper” mutation has been shown to confer resistance to BRAF inhibition when introduced into BRAF mutant cell lines and in genetically engineered mouse models [38]. Therefore, while it is possible that secondary BRAF mutations may be identified as more tumors with acquired resistance to BRAF inhibitors are analyzed, it does not appear that secondary BRAF mutations are a common cause of acquired BRAF inhibitor resistance in the clinic.

### Amplification of mutant BRAF

While secondary mutations in BRAF have not been identified as a cause of BRAF or MEK inhibitor resistance, our laboratory recently identified selective amplification of the mutant BRAF allele as the mechanism underlying acquired resistance in two independent BRAF mutant colorectal cancer cell lines selected for resistance to the MEK inhibitor AZD6244 [39]. BRAF amplification was also recently identified by another laboratory as the mechanism of acquired resistance to MEK inhibitors in a different BRAF mutant colorectal cell line model, corroborating these findings [40]. Resistant BRAF-amplified clones were also cross-resistant to BRAF inhibitors, although to a slightly lesser degree. Surprisingly, even though MEK inhibitors act downstream of BRAF, BRAF gene amplification dramatically reduced the ability of AZD6244 to inhibit ERK phosphorylation and, as a consequence, to inhibit cell proliferation and survival. Although BRAF amplification arose as an acquired resistance mechanism in vitro, BRAF amplification has not been identified as a mechanism of acquired resistance in clinical samples, given that few MEK inhibitor-resistant tumor biopsies are available. However, we identified pre-existing BRAF amplification in a treatment-naive BRAF mutant colorectal cancer, suggesting that BRAF amplification could also be a cause of *de novo* resistance to BRAF and MEK inhibitors in the clinic.

We found that the mechanism by which BRAF amplification led to BRAF and MEK inhibitor resistance hinged upon hyperactivation of MEK. We observed that the levels of phosphorylated MEK (P-MEK) in resistant cells were 5 to 6 times higher than the basal levels seen in parental cells. Careful evaluation of the dose-response relationship between BRAF inhibitor treatment and phosophorylation of MEK and ERK revealed that, in resistant cells, levels of P-MEK could be reduced by ~50% before any noticeable decrease in P-ERK levels was observed. This was in stark contrast to parental cells, in which a ~50% decrease in P-MEK levels led to a ~50% decrease in P-ERK levels. These findings suggested that the high levels of P-MEK in resistant cells (driven by BRAF amplification) were in excess of levels required for near-maximal ERK phosphorylation. As a result, a much higher concentration of BRAF or MEK inhibitor was required to fully suppress ERK phosphorylation in resistant cells, either by reducing excess P-MEK levels (as in the case of the BRAF inhibitor) or by inhibiting excess MEK activity (as in the case of the MEK inhibitor). However, if resistant cells were treated with a low dose of BRAF inhibitor sufficient to reduce levels of P-MEK to amounts observed under basal conditions in parental cells, the ability of MEK inhibitors to suppress P-ERK was completely restored. Accordingly, while resistant cells were insensitive to BRAF or MEK inhibitors individually, combined BRAF and MEK inhibition fully overcame resistance and induced dramatic apoptosis and growth inhibition in these cells. Furthermore, combined BRAF and MEK inhibition was also more effective in parental cells, suggesting a possible broader utility for combinatorial targeting of the RAF-MEK pathway in BRAF mutant cancers.

This mechanism underlying the resistance to BRAF and MEK inhibitors caused by BRAF amplification has potential implications for other models of resistance in BRAF mutant tumors. Since excess levels of activated and phosphoryated MEK underlie the mechanism of resistance to BRAF and MEK inhibitors, it is possible that other changes that lead to similar degrees of MEK hyperactivation could cause a similar mode of resistance. For example, excessive upstream input from receptor tyrosine kinases (RTKs), RAS or RAF proteins, or other activators of MEK, could also potentially lead to MEK hyperactivation and result in similar resistance to BRAF or MEK inhibitors.

### Elevated CRAF activity

Montagut et al identified elevated CRAF activity as a mechanism of resistance to the BRAF inhibitor AZ628 in pre-clinical studies [41]. In AZ628-resistant clones generated in vitro from a BRAF V600 mutant melanoma cell line, P-ERK levels were maintained despite treatment with the inhibitor. Elevated CRAF protein levels were present in resistant clones, relative to drug-sensitive parental cells, whereas levels of ARAF and BRAF were unchanged. No CRAF gene amplification and no increase in CRAF transcript were noted, suggesting that elevated CRAF levels arose from a post-transcriptional mechanism. In this model, tumor cells appear to have switched their dependency from BRAF to CRAF. Thus, resistant clones were sensitive to CRAF knockdown or to Hsp90 inhibitors, which down-regulated CRAF protein levels. CRAF overexpression in parental cells also produced AZ628 resistance. Interestingly, resistant clones with elevated CRAF levels retained some sensitivity to MEK inhibitors, although with reduced potency.

### Activating NRAS mutation

Nazarian et al recently identified NRAS mutations as a mechanism of acquired resistance to the BRAF inhibitor PLX4032 [37]. NRAS mutations are present in 15-30% of melanomas, but are rarely coincident with BRAF mutations [42, 43]. Cell lines resistant to PLX4032 were derived from three melanoma cell lines with BRAF mutations. In one of these cell lines, an NRAS Q61K mutation was identified. An NRAS Q61K mutation was also identified in an isolated nodal metastasis from a patient with BRAF mutant melanoma, which progressed after an initial response to PLX4032. Interestingly, a distinct NRAS mutation (Q61R) was identified in a second progression site in the same patient. In resistant cells in vitro, both P-MEK and P-ERK levels were maintained despite the presence of BRAF inhibitor. It is therefore likely that mutant NRAS leads to activation of MEK by signaling through RAF isoforms other than BRAF. However, both the PLX4032-resistant cell line and a short-term culture line from the above patient's resistant disease focus—each harboring an acquired NRAS mutation—retained sensitivity to MEK inhibitor alone and to the combination of PLX4032 and a MEK inhibitor. Interestingly, in early clinical trials with MEK inhibitors in unselected patient populations, responses to single agent MEK inhibitor were observed in patients with NRAS mutant melanomas, including one complete response [15]. These data suggest that MEK inhibitors or a RAF/MEK inhibitor combination could be a potential therapy to overcome NRAS-mediated resistance to BRAF inhibitors.

### Increased levels of COT/Tpl2

Johannessen et al used an ORF expression library encoding approximately three-quarters of the human “kinome” to identify kinases that confer resistance to the BRAF inhibitor PLX4720 when they are overexpressed in sensitive BRAF V600E melanoma cell lines [44]. Nine kinases were identified, two of which caused P-MEK and P-ERK levels to be maintained despite the presence of PLX4720. These two kinases were CRAF (consistent with the findings of Montagut et al, above [41]) and COT/Tpl2, encoded by *MAP3K8*. These data suggest that activation of MEK-ERK signaling by COT represents a novel RAF-independent mechanism of MEK activation. Interestingly, COT levels were observed to increase in cell lines treated with BRAF inhibitors, suggesting that COT may be involved with feedback regulation of MEK activity. Consistent with these findings, in the biopsies of two of three patients taken during treatment with PLX4032, COT transcript levels were elevated relative to pre-treatment biopsies. In one patient who was biopsied post-relapse, levels of COT transcript were elevated relative to pre-treatment and on-treatment biopsies, suggesting that COT may contribute to acquired resistance to BRAF inhibitors in the clinic. The authors also identified two cell lines with copy number gains at the *MAP3K8* locus that expressed high levels of COT. These cell lines were resistant to PLX4720. Interestingly, even though COT appears to activate ERK through MEK, these cell lines and cell lines overxpressing exogenous COT were also resistant to MEK inhibitors. One possible explanation for this observation is that high COT levels might contribute to MEK inhibitor resistance by causing MEK hyperactivation, similar to the mechanism observed for BRAF amplification [39]. Accordingly, combined BRAF and MEK inhibition was able to overcome resistance caused by elevated COT levels.

### ERK-independent mechanisms of acquired resistance

As discussed above, the majority of the resistance mechanisms to BRAF and MEK inhibition that have been identified lead to ERK reactivation and retain dependence on ERK signaling. While this underscores the importance of the RAF-MEK-ERK pathway in melanoma proliferation and survival, in the last few months several examples of resistance mechanisms have been reported that do not rely on sustained ERK signaling, indicating that other “ERK-independent” pathways can compensate for loss of ERK activity and can maintain the tumorigenicity of BRAF V600E melanoma in the absence of ERK activation.

### PDGFRβ overexpression

In the recent study by Nazarian et al, discussed above, of the three BRAF mutant melanoma cell lines made resistant to PLX4032, one resistant cell line was found to have acquired an NRAS mutation and maintained P-ERK levels despite the presence of BRAF inhibitor [37]. In the other two models, PLX4032 inhibited ERK phosphorylation to a similar degree as it did in the parental cell lines. The ability of these resistant cell line models to proliferate and survive despite suppression of P-ERK by PLX4032 suggested activation of “ERK-independent” proliferation and survival signals. Consistent with the lack of dependence on the ERK pathway demonstrated by these resistant cell line models, treatment with a MEK inhibitor or combined MEK and BRAF inhibition failed to overcome resistance, as it did with the ERK-dependent models discussed above. Comparison of the microarray gene expression profiles of parental and resistant cells revealed overexpression of several RTKs in the resistant cells, including KIT, MET, EGFR and PDGFRβ. Of these four RTKs, only EGFR and PDGFRβ showed increased protein expression in the resistant cell lines, and only PDGFRβ displayed increased activation-associated tyrosine phosphorylation in resistant cells. Importantly, the authors found that four of eleven clinical post-relapse biopsies from melanoma patients treated with PLX4032 showed increased PDGFRβ expression, relative to pre-treatment biopsies. To validate PDGFRβ as the cause of resistance in their cell line models, the authors demonstrated that RNAi-mediated knockdown of PDGFRβ in resistant cells led to growth inhibition in the presence of PLX4032. However, PDGFRβ knockdown did not restore an apoptotic response in these cells in the presence of PLX4032, suggesting that PDGFRβ overexpression may not be the only mechanism of resistance in these cells. Consistent with this possibility, the combination of the PDGFRβ inhibitor imatinib and PLX4032 did not restore sensitivity to resistant cell lines. Therefore, it is conceivable that an additional mechanism could be contributing to resistance, possibly involving one of the other RTKs whose expression was increased in resistant cells. Still, this model of resistance demonstrates that BRAF or MEK inhibitor resistance can arise in the absence of ERK reactivation and that RTKs may promote resistance through activation of ERK-independent proliferation and survival pathways.

### IGF1R activation

Villanueva et al recently identified another RTK-driven resistance mechanism through in vitro modeling of BRAF inhibitor resistance in BRAF V600E melanoma cell lines [45]. In cell lines made resistant to the BRAF inhibitor SB-590885, P-ERK was no longer suppressed by BRAF inhibition, suggesting activation of MEK-ERK signaling through another RAF isoform. Interestingly, knockdown of individual RAF isoforms revealed that no dominant RAF isoform controlled MEK-ERK signaling in resistant cells, unlike the situation observed with CRAF by Montagut et al [41]. Rather, P-ERK could only be suppressed if all three RAF isoforms were inhibited simultaneously. Alternatively, MEK inhibition was also capable of blocking ERK phosphorylation. These findings suggest that ERK is still activated in a MEK-dependent manner in the resistant cells, but that activation of MEK can proceed through any of the three RAF isoforms, consistent with activation of an upstream activator of RAF signaling. However, despite complete inhibition of P-ERK, MEK inhibition produced only cytostatic effects on resistant cells and failed to induce apoptosis, as it did in parental cells, suggesting activation of an ERK-dependent survival pathway in the resistant cells.

Because RTKs can signal through multiple RAF isoforms by activation of RAS proteins, and since RTKs activate multiple signaling pathways in addition to RAF-MEK-ERK, the authors investigated whether resistant cells showed differences in RTK phosphorylation relative to parental cells. More than one RTK exhibiting differential phosphorylation was identified by phospho-RTK array analysis, including IGF1R. However, pharmacologic inhibition of IGF1R decreased proliferation of resistant cells, and combined inhibition of IGF1R and MEK induced dramatic apoptosis, suggesting that ERK-independent survival signaling was mediated by IGF1R in resistant cells. While surface expression of IGF1R was found to be increased in resistant cells, the exact mechanism leading to increased IGF1R activation was not identified. In addition to activating the RAF-MEK-ERK pathway, IGF1R and other RTKs are known to activate PI3K-AKT signaling, which is known to be an important regulator of cell survival and proliferation [46, 47]. Resistant cells displayed elevated levels of phosphorylated AKT (P-AKT) compared to parental cells, and IGF1R inhibition could reduce P-AKT levels in resistant cells. Combined pharmacologic inhibition of PI3K and MEK was also able to induce apoptosis in resistant cells, indicating that the PI3K-AKT pathway mediated ERK-independent survival signals in these resistant cell line models.

Taken together, these two models of resistance driven by RTKs indicate that RTK-mediated resistance to BRAF or MEK inhibition highlights the complexity of signaling in resistant cells. Although in each resistance model, a dominant RTK was identified (e.g. PDGFRβ or IGF1R) that contributed to the majority of the signaling changes and to the decrease in drug sensitivity observed in resistant cells, there was evidence in each model that additional signaling pathways, perhaps involving other RTKs, were contributing to resistance. First, in each model, multiple RTKs were found to be elevated or to display increased phosphorylation, suggesting that other RTKs could contribute in some way to resistance. Second, in each model, inhibition of the dominant RTK, either using pharmacologic inhibitors or RNAi-mediated knockdown, was not sufficient to completely reverse all of the changes in signaling and sensitivity seen in the resistant cells.

In the study by Nazarian et al, PDGFRβ knockdown blocked proliferation, but failed to induce apoptosis, even in the presence of BRAF inhibitor [37]. Furthermore, pharmacologic inhibition of PDGFRβ with imatinib did not restore sensitivity of resistant cells in the presence or absence of BRAF inhibitor. In the study by Villanueva et al, pharmacologic inhibition of IGF1R in combination with MEK was able to restore an apoptotic response in resistant cells [45]. However, inhibition of IGF1R could not restore the ability of BRAF inhibitor to suppress P-ERK, and, as a result, the addition of BRAF inhibitor did not lead to a greater reduction in cell viability compared to IGF1R inhibition alone. These findings suggest that the signaling through multiple RAF isoforms to MEK observed in resistant cells may not be due to increased IGF1R activation, but rather may involve other signals, perhaps from one of the other RTKs (e.g. MET) noted to demonstrate increased phosphorylation in their analysis of resistant cells. The complexity of each of these RTK-driven resistance models indicates that both the identification and subsequent targeting of the responsible RTK may be challenging in patients with BRAF mutant cancers who relapse while on treatment with BRAF or MEK inhibitors.

One potential strategy to overcome this problem would be to target common signaling nodes activated by RTKs in resistant cells, rather than attempting to target specific RTKs. In addition to activating RAF-MEK signaling, the PI3K-AKT pathway is a major signaling output of RTKs, and several studies have shown that inhibiting PI3K signaling in combination with RAF-MEK signaling is sufficient to induce apoptosis and suppress proliferation in RTK-driven cancer models [48-50]. Accordingly, as demonstrated by Villanueva et al, treatment of resistant cells with increased IGF1R activation with the combination of PI3K and MEK inhibitors restored an apoptotic response [45]. While this inhibitor combination was not tested in the PDGFRβ-driven resistance model discussed above, one might predict that combined PI3K and RAF-MEK inhibition would overcome resistance in this model as well. In support of this hypothesis, another in vitro model of BRAF inhibitor resistance in BRAF mutant melanoma was recently reported by Jiang et al [51]. While the exact mechanism of resistance was not identified, the mechanism appeared highly dependent on extracellular signals and serum concentration, and resistant cells showed increased levels of P-AKT, suggesting that activation of PI3K signaling, perhaps by RTKs, could also be involved in promoting resistance. In this model, treatment of resistant cells with the combination of a PI3K inhibitor and a BRAF inhibitor was able to overcome resistance. Collectively, these results suggest that co-targeting the PI3K and RAF-MEK pathways could constitute a potential strategy to overcome ERK-independent mechanisms of BRAF or MEK inhibitor resistance, including RTK-driven resistance.

## IMPLICATIONS AND THERAPEUTIC STRATEGIES

While the recent clinical successes of BRAF and MEK inhibitors in BRAF mutant cancers are encouraging, many of the responses to therapy have been short-lived due to rapid development of acquired resistance [16]. As a result, there is an urgent clinical need for therapeutic strategies for patients with BRAF mutant cancers who eventually progress on BRAF or MEK inhibitor therapy. The in vitro resistance models reviewed herein suggest that the most appropriate choice of therapy for patients with recurrent disease may depend on whether that particular patient's resistant tumor is driven by an ERK-dependent or ERK-independent mechanism. As shown in Table 1, ERK-dependent resistant models retain sensitivity to combined treatment with BRAF and MEK inhibitors. It is important to note that, while the combination of BRAF and MEK inhibitors was not tested in resistant cells harboring elevated CRAF levels, these resistant cells retained sensitivity to single agent MEK inhibitor, suggesting that they would also be sensitive to combined BRAF and MEK inhibition [41]. Conversely, combined BRAF and MEK inhibition was not effective when tested in ERK-independent resistance models, presumably due to the activation of alternative proliferation and survival pathways outside of the RAF-MEK axis. Instead, combined inhibition of PI3K and MEK or PI3K and BRAF was effective in the ERK-independent models in which it was tested. Thus, a reasonable initial clinical strategy for patients who relapse on single-agent BRAF or MEK inhibitor would be to treat with the combination of a BRAF and a MEK inhibitor if their tumor harbors an ERK-dependent resistance mechanism or to treat with the combination of a PI3K inhibitor and a MEK or BRAF inhibitor if their resistant tumor is driven by an ERK-independent mechanism. Since each of these drug combinations is currently being evaluated in clinical trials, with similar combination trials being planned, it is feasible that this basic strategy could be implemented at the present time [52]. In addition, newer agents in development, such as ERK inhibitors, may also play a role in therapeutic approaches to overcome resistance in the future.

**Table 1:** Inhibitor sensitivity profiles of resistant BRAF mutant cell line models The sensitivity of each resistant cell line model to BRAF inhibitor alone (BRAF), MEK inhibitor alone (MEK), the combination of a BRAF and MEK inhibitor (BRAF+MEK), and the combination of a PI3K inhibitor and either a RAF or MEK inhibitor (PI3K+RAF/MEK) is shown. For each condition, resistant cell line models are designated as sensitive (+), insensitive (-), or not tested (NT). Inhibition of proliferation without induction of apoptosis is designated as (+/−).

			Sensitivity to Inhibitors			
	Mechanism	Study	BRAF	MEK	BRAF + MEK	PI3K + RAF/MEK
**ERK-Dependent**	MEK1 mutation	Emery et al, 2009 [35]	−	−	+	NT
	BRAF amplification	Corcoran et al, 2010 [39]	−	−	+	NT
	CRAF elevation	Montagut et al, 2008 [41]	−	+	NT	NT
	NRAS mutation	Nazarian et al, 2010 [37]	−	+	+	NT
	COT elevation	Johannessen et al 2010 [44]	−	−	+	NT
**ERK-Independent**	PDGFRβoverexpression	Nazarian et al, 2010 [37]	−	−	−	NT
	IGF1R activation	Villaneuva et al, 2010 [45]	−	+/−	NT	+
	Unidentified, ?PI3K	Jiang et al, 2010 [51]	−	−	−	+

However, in order to apply this basic therapeutic strategy most effectively, it would first be necessary to identify whether the mechanism driving a given patient's resistant tumor is ERK-dependent or ERK-independent. For this reason, routinely obtaining biopsies from recurrent tumors in patients treated with BRAF or MEK inhibitors will likely be important for the selection of the most appropriate therapy post-relapse, as has been done for acquired resistance to other targeted therapies [53]. Particularly as the frequencies of specific resistance mechanisms in BRAF mutant cancers become better understood, focused analysis of biopsies from resistant tumor foci for common resistance mechanisms might allow identification of the cause of drug resistance and could guide second-line therapy.

Still, while it might be feasible to screen tissue from resistant tumors for a small set of the most common individual alterations leading to resistance, the above studies demonstrate that resistance can arise through numerous specific molecular events. Therefore, especially as more specific mechanisms of resistance are defined, it may become unreasonable or even impossible to identify the specific change driving tumor resistance in every patient. As a result, an alternative approach might be to assess resistant tumor specimens for common indicators of ERK-dependent or ERK-independent resistance, perhaps by assessing biomarkers of RAF-MEK-ERK or PI3K-AKT signaling in a biopsy taken while the patient remains on treatment. For example, if P-ERK levels remain suppressed in a resistant tumor biopsy taken in a patient who remains on therapy (such as seen in the PDGFRβ-driven model of Nazarian et al [37]), this finding would indicate an ERK-independent mechanism, since all ERK-dependent mechanisms restore ERK phosphorylation despite the presence of inhibitor. Lack of P-ERK signal would suggest that this patient would best be treated with the combination of a PI3K inhibitor and a MEK or BRAF inhibitor. However, the presence of P-ERK in a resistant tumor biopsy does not guarantee an ERK-dependent mechanism, and thus it would not be possible to determine based on the presence of P-ERK alone whether a patient would benefit more from the combination of a MEK and a BRAF inhibitor or the combination of a PI3K inhibitor and a BRAF or MEK inhibitor. For example, in the RTK-driven resistance model developed by Villanueva et al, in addition to the activation of an ERK-independent survival pathway PI3K via IGF1R, ERK phosphorylation was also restored in the presence of BRAF inhibitor, likely due to RTK-driven signals through other RAF isoforms [45]. Therefore, to select the optimal combination strategy, it might be useful to assess markers of PI3K signaling in addition to evaluating P-ERK levels. In fact, both this resistant model and clinical biopsies from resistant tumor with increased IGF1R activation showed elevated P-AKT levels. Therefore, while combined BRAF and MEK inhibition may be a reasonable default treatment for patients with clinically acquired BRAF or MEK inhibitor resistance, given that most resistance mechanisms identified to date involve ERK-dependent mechanisms, lack of ERK reactivation or the presence of increased P-AKT levels could indicate cases in which use of a PI3K and a MEK or BRAF inhibitor might be more effective.

Finally, it is intriguing to speculate that anticipation of acquired resistance to BRAF or MEK inhibitors could lead to strategies to prevent resistance from emerging. Moreover, since it is possible for multiple distinct resistance mechanisms to arise in the same patient, employing combination strategies aimed at preventing resistance as part of a patient's initial therapy could have advantages. Recent studies have suggested that combined BRAF and MEK inhibition is more effective in treatment-naïve BRAF mutant cancers than treatment with either inhibitor alone [39, 54]. Furthermore, one study showed that initial treatment with the combination of a BRAF and MEK inhibitors can prevent or delay emergence of resistance due to ERK pathway reactivation in BRAF mutant melanomas [54]. Similarly, initial combined inhibition of AKT and MEK was also shown to prevent resistance in BRAF mutant melanoma cell lines that develop resistance to MEK inhibitors through upregulation of PI3K-AKT signaling [55]. As a result, clinical trials assessing combinations of targeted inhibitors for the initial treatment of BRAF mutant cancers are currently underway [52]. Thus, identification and understanding of the mechanisms of resistance to BRAF or MEK inhibitors in BRAF mutant cancers could not only lead to strategies to overcome established resistance, but may yield a means by which to prevent resistance from emerging and to prolong the clinical response to therapy.
